# Introducing rotavirus vaccine in the Universal Immunization Programme in India: From evidence to policy to implementation

**DOI:** 10.1016/j.vaccine.2019.07.104

**Published:** 2019-09-16

**Authors:** Akash Malik, Pradeep Haldar, Arindam Ray, Anita Shet, Bhrigu Kapuria, Sheenu Bhadana, Mathuram Santosham, Raj Shankar Ghosh, Robert Steinglass, Rakesh Kumar

**Affiliations:** a United Nations Development Programme, India; b Ministry of Health and Family Welfare, Government of India, India; c Bill & Melinda Gates Foundation, India; d International Vaccine Access Center, Johns Hopkins Bloomberg School of Public Health, Baltimore, USA; e UNICEF, India; f Immunization Technical Support Unit, Ministry of Health and Family Welfare, Government of India, India; g John Snow Inc., Arlington, VA, USA

**Keywords:** Rotavirus vaccine, Diarrhea, Immunization

## Abstract

**Background:**

In 2016, India became one of the first countries in Asia to introduce an indigenously manufactured rotavirus vaccine. However, any new vaccine introduction needs to be meticulously planned to allow for strengthening of the existing immunization systems instead of burdening them.

**Methods:**

The process of rotavirus vaccine introduction in India started with the establishment of National Rotavirus Surveillance Network in 2005 which generated relevant evidence to inform policy level decisions to introduce the vaccine. The preparatory activities started with assessment of health systems and closing any gaps. This was followed by development of vaccine specific training packages and cascade training for programme managers and health workers. The introduction was complemented with strong communications systems and media involvement to allow for good acceptability of the vaccine on the ground. Each step of introduction was led by the government and technically supported by development partners.

**Results:**

India introduced rotavirus vaccine in a phased wise manner. In the first two phases the vaccine has been introduced in nine states of the country accounting for nearly 35% of the annual birth cohort of the country. From March 2016 to November 2017, approximately 13,260,000 rotavirus vaccine doses were administered in the country. The vaccine was well accepted by both the health workers and parents/caregivers.

**Conclusion:**

Rotavirus vaccine introduction in India is an excellent example of how government stewardship with well-defined roles for development partners can allow a new vaccine introduction to be used as a system strengthening activity.

## Highlights

• India became one of the first countries in Asia to introduce rotavirus vaccine.
• Rotavirus vaccine is being expanded to the entire country in a phase wised manner.
• The new vaccine introduction strengthened the programme rather than burdening it.

## 1 Introduction

India has come a long way in reducing mortality among infants and children under five years. The infant mortality rate declined by almost 28% from 47 per thousand live births in 2010 to 33 per thousand live births in 2017 [1], [2]. Similarly, the under-5 mortality rate has declined by 29% from 55 per thousand live births in 2011 to 39 per thousand live births in 2016 [3]. Despite these impressive declines in the mortality rate among infants and children, diarrheal diseases remain the leading cause of childhood mortality globally as well as in India. Diarrhea is responsible for about 9% of under-five deaths globally and 10% under-five deaths in India causing around 110,000 deaths annually [4]. Diarrhea is caused by various types of bacteria and virus but among these, rotavirus (RV) diarrhea is the most common cause of diarrhea mortality and morbidity among children under five years of age. Rotavirus diarrhea is responsible for 29% of all diarrhea related deaths globally and 40% of moderate and severe diarrheal episodes in India [5]. Diarrhea is also an important contributor to long-term nutritional deficiency complications like stunting, wasting, malnutrition and loss of cognitive development potential.

India’s Universal Immunization Programme (UIP) has been progressing at a commendable pace. After 2010, the introduction of new vaccines and a focus on strengthening the routine immunization system has gained momentum. In the last decade the spectrum of diseases covered under the programme has seen the addition of several new antigens like monovalent Japanese encephalitis; hepatitis B and *Haemophilus influenzae* type b as part of the combined pentavalent vaccine; rubella as part of combined measles-rubella vaccine used in campaigns; pneumococcal conjugate vaccine; and inactivated polio vaccine as part of the polio endgame strategy. With India being declared in 2014 as ‘polio free’ and validated for maternal and neonatal tetanus elimination in 2015, confidence in new vaccines has risen considerably. One of the most common vaccine-preventable disease with high mortality in children is RV diarrhea. On 26th March 2016, India became the first country in WHO’s South East Asia region to introduce rotavirus vaccine (RVV). The first two phases of RVV introduction covering more than 35% of the annual birth cohort were government funded, with technical assistance from development partners. The aim of this manuscript is to document how research has generated evidence to inform policy which has been translated into a successful public health programme with respect to RVV introduction.

## 2 Approach

### 2.1 Research to evidence

#### 2.1.1 Availability of indigenous vaccine products

Out of four licensed RVVs in India, Rotateq® and Rotarix® are manufactured outside India while Rotavac® and Rotasiil® were developed and are being manufactured in India. All the four vaccines have been shown to be safe, effective and efficacious in various trials done around the world [6], [7], [8], [9]. While Rotavac® vaccine is currently being used in nine states in India, Rotasiil® vaccine is being introduced in Jharkhand. The availability of four RVV products has allowed for vaccine security and competitive pricing for a large country like India.

#### 2.1.2 Establishment of national rotavirus surveillance network

The Ministry of Health and Family Welfare, Government of India (MoHFW, GoI) and the Indian Council of Medical Research (ICMR) in December 2005, established the National Rotavirus Surveillance Network (NRSN) to estimate and monitor RV disease burden in children <5 years hospitalized for diarrhea [10].

The first round of NRSN (NRSN-I) was conducted between 2005 and 2009 with four laboratories and ten hospitals in six different states of India. The surveillance detected RV in 39% of children admitted for diarrhea. The circulation of a diverse range of RV strains was also established with G2P [4] being the most common type, consisting of 25.7% of strains, followed by G1P [8] (22.1%), and G9P [8] (8.5%). These findings demonstrated the need for preventative measures including improvement in environmental sanitation, water supply and food hygiene in addition to advocacy for the introduction of RVV into the national programme [10].

NRSN-1 was expanded in 2012 to include four referral and seven regional laboratories in the Southern, Western, Northern and Eastern regions of the country, and 28 clinical recruitment sites in 17 states and two Union Territories. The findings in the expanded NRSN corroborated those of NRSN-1: between September 2012 and December 2014, RV was detected in 39.6% of 10,207 children hospitalized with acute gastroenteritis. Infections were more commonly seen among younger children and most of the infected children had severe or very severe disease [11].

J John et al, in 2014 came up with conservative estimates on burden of RV diarrhea in India by combining the data and statistics from various available sources and organizations namely, NRSN (2005–2009), Million Death Study (MDS), World Health Organization (WHO) and UNICEF with data from five community-based cohorts. These estimates showed that RV diarrhea was responsible for nearly 78,000 deaths, 0.87 million hospitalizations, 3.27 million out-patient visits and 11 million episodes in children less than 5 year of age every year [12].

This national level surveillance established the public health importance of RV diarrhea in India and reiterated the need for the RV vaccination. The region- and state-wise RV burden data generated by NRSN convinced policy makers at state and national level about the high disease burden caused by RV and the need to incorporate the vaccine into the UIP.

### 2.2 Evidence to policy

While the NRSN established the burden of RV diarrhea in India, there are several other general considerations for introduction of new vaccines into the national immunization programme of any country. Among others, these include political and public health priorities; effectiveness of other public health measures to prevent and control the disease; financial stability of the immunization programme; and alignment with global priorities and goals.

**Fig. 1:**
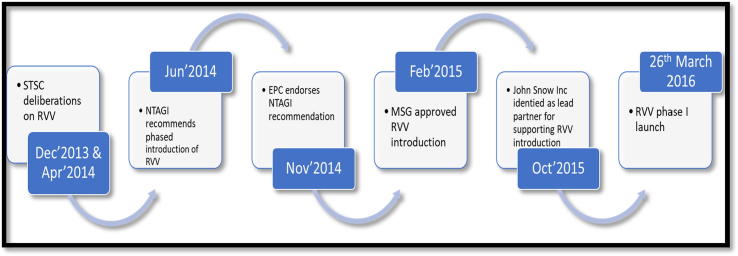
Decision making process for RVV introduction in India.

An informed decision-making process was already in place in India to assess and implement the introduction of RVV in India. An independent technical body, National Technical Advisory Group on Immunization (NTAGI), in June 2014 recommended a phased introduction of RVV based on Standing Technical Sub Committee (STSC) meeting deliberations [13]. The STSC deliberated on evidence for safety, efficacy, side effects, cost-effectiveness, affordability of available RVVs, programme capacity, and effectiveness of alternative/complementary measures of diarrhea control in India. In November 2014 the Empowered Programmee Committee (EPC) endorsed the NTAGI recommendation followed by approval by Government of India’s Mission Steering Group (MSG) of National Health Mission in February 2015 (Fig. 1). In its first phase the RVV was introduced in the four states of Haryana, Himachal Pradesh, Andhra Pradesh and Odisha, which accounted for nearly 9% of the birth cohort of the country.

**Fig. 2:**
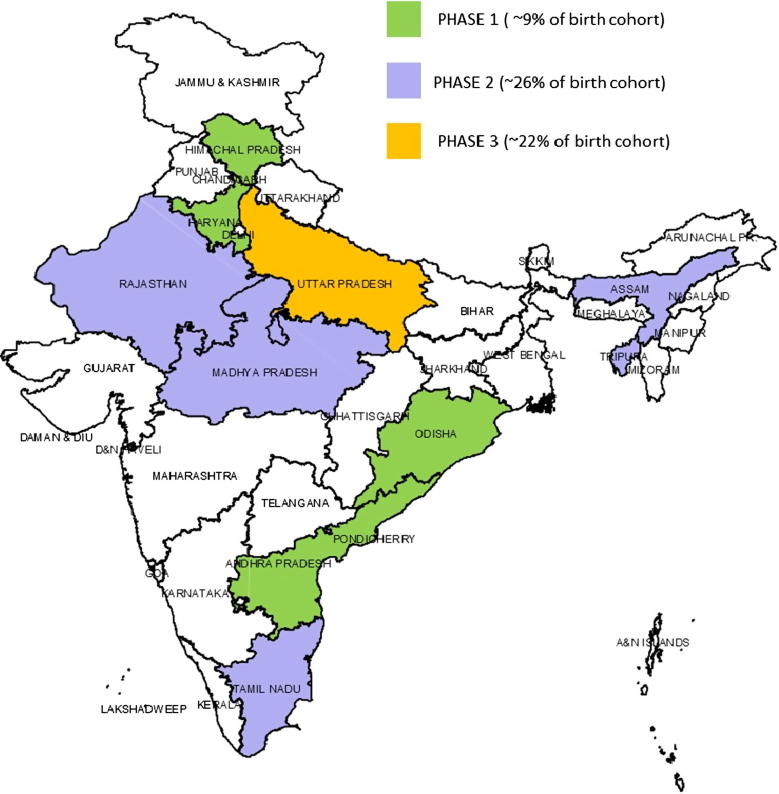
RVV introduction in India – Phase 1 and 2 states.

Phase 2 of RVV introduction included the four states of Rajasthan, Assam, Madhya Pradesh, and Tamil Nadu (Fig. 2). Phase 2 commenced only after a detailed review of Phase 1 by the ICMR. The Phase 2 states accounted for a total of nearly 26% of the birth cohort of the country. The selection of the states for each of the phases was done based on the burden of RV diarrhea according to NRSN data, as well as the ability and readiness of the state health system to take up new vaccine based on past experience.

#### 2.2.1 Status and effectiveness of the other public health measures to prevent and control the disease (Fig. 3)

The Government of India introduced the National Diarrheal Disease Control (NDDC) Programme in 1978 to address the burden of diarrheal disease. In 1992–93 the programme was made part of the Child Survival and Safe Motherhood (CSSM) strategy and later incorporated into the National Rural Health Mission (NRHM) in 2005 [14], [15]. The diarrhea control programme was further strengthened by the addition of zinc (10 mg elemental zinc for infants 2 to 6 months and 20 mg/day for children >6 months for 14 days) for children 3 months and above and vitamin A for all children from 9 months to 5 years of age [16]. In 2013, the Ministry of Health and Family Welfare, Government of India (MoHFW, GoI) issued the Infant and Young Children Feeding (IYCF) guidelines bringing greater attention and commitment to promote IYCF interventions at the health facility, community and household levels [17]. Since 2014, India has been implementing the Intensified Diarrhea Control Fortnight (IDCF) with an aim of achieving improved coverage of essential life-saving commodities of oral rehydration solution (ORS) and zinc dispersible tablets and increasing the practice of appropriate child feeding during diarrhea. The programme also focuses on demonstration of correct handwashing techniques in schools and establishment of IYCF demonstration and counselling centers [18].

**Fig. 3:**
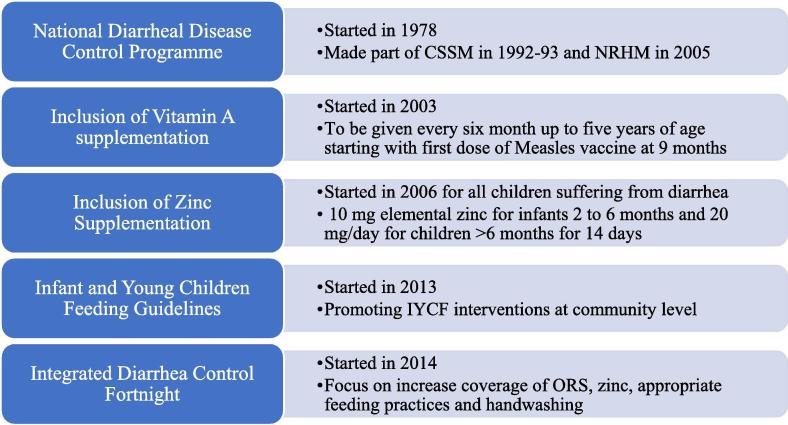
Government initiatives to reduce the burden of diarrhea in India apart from and prior to RVV introduction.

Although the GoI has introduced several public health interventions to reduce morbidity and mortality due to diarrhea in under-five children, diarrhea continues to be the second largest cause of under-five mortality in the country. In addition, since the most common cause of diarrhea in infants is RV, none of the above interventions especially targeted RV infection since vaccination is the only known specific preventive measure to curb this particular cause of acute gastroenteritis.

#### 2.2.2 The cost effectiveness of the vaccination programme vis-à-vis alternatives other than vaccination

Various studies have established that RV vaccination is a cost-effective intervention to prevent childhood morbidity and mortality against RV diarrhea. In their impact and cost effectiveness analysis in India, Rheingans R et al found that RV vaccination was very cost effective (cost-effectiveness ratio; $105-$298/DALY averted) even with the cost per dose as high USD 7.5 [19]. The authors assumed the efficacy of 50% for full course with RVV to allow for the model to be more generalized instead of focusing on a particular RVV product. In their publication on cost effectiveness of RV vaccination in India and Ethiopia, Verguet et al estimated that RV vaccination can help avert $1,800,000 household expenditure on diarrhea in India, if a two dose RVV was used (costing about USD 5.5 per dose) This was further expected to increase with reduced vaccine prices (target price of USD 2) in presence of GAVI co-financing for India [20]. A recent study on return of investment of various antigens in low- and middle-income countries by Ozawa et al, found that RV vaccination was a good return on investment (RoI of 1.3) and this was expected to increase with further reduction in vaccine prices [21]. RVV has thus been shown to be cost effective in India and other Middle- and Low-income countries. It is worth noting that the choice of RV vaccine products considered for the above-mentioned studies differs in some cases from the RV vaccines being introduced in India and further that with the availability of additional RV vaccine products, some locally manufactured in India at a lower purchase price, the impact and cost-effectiveness is expected to be even greater.

#### 2.2.3 Financial sustainability of the vaccination programme

The first two phases of RVV introduction (Fig. 2) covering 35% of the annual birth cohort was fully funded by the GoI. This included the costs of vaccine procurement, training of health workers, development and printing of communication material, strengthening of cold chain capacity and surveillance for adverse events following immunization (AEFI). The immunization partners like WHO, John Snow Inc. (JSI), UNICEF, Global Health Strategies (GHS) and PATH provided catalytic technical support with expertise in developing training materials, serving as master trainers of trainers (ToT), and supporting monitoring and evaluation of the roll out. Assessment of expenditures incurred on RVV introduction is beyond the scope of this paper. However, India would have additionally required an estimated USD 50 million if the vaccine had been introduced country wide in 2016, 90% of which would have been borne by the GoI as with the rest of the immunization programme. The largest share of this total cost (nearly 38%) was attributed to the hours spent by health officers and field-level health workers in implementation of the immunization programme as a whole [22].

The third phase of RVV introduction in Uttar Pradesh has been initiated since July 2018 with the vaccine procured through support from Gavi, the Vaccine Alliance. However, the implementation and roll out of vaccination is being funded by MoHFW, GoI. Currently, the MoHFW, GoI aims to expand RVV to the entire country by the end of 2019, and the expansion will be completely funded by the government with additional technical support from partner agencies.

#### 2.2.4 Public health and political priorities: alignment with global and regional recommendations

In its position paper for RV, the WHO recommended that “Rotavirus vaccines should be included in all national immunization programmes and considered a priority, particularly in countries with high RV gastroenteritis-associated fatality rates, such as in south and south-eastern Asia and sub-Saharan Africa.” The age for first dose of RV vaccine was recommended to be 6 weeks along with first dose of diphtheria, tetanus and pertussis (DTP). It was further reiterated that “the benefits of rotavirus vaccination against severe diarrhea and death from rotavirus infection far exceeds the risk of intussusception [23].”

As a key strategy to prevent RV diarrheal morbidity and mortality, the Global Action Plan for Prevention of Diarrhea and Pneumonia (GAPPD) also emphasizes the importance of RVV along with exclusive breastfeeding, vitamin A supplementation, complementary feeding, hand washing and clean water and sanitation [24].

Thus, RVV introduction in India was guided by a strong global commitment to tackle morbidity and mortality due to diarrhea and a thorough review of the existing evidence by the GoI NTAGI.

### 2.3 Policy to programme

#### 2.3.1 Immunization programme in India

India’s UIP is one of the largest immunization programmes in the world with an annual target of 26.5 million infants and 30 million pregnant women. The programme reaches the target population through more than 27,000 cold chain points and approximately nine million vaccination sessions every year. The UIP is guided by the National Health Policy 2017 and the National Vaccine Policy 2011 which strive to further increase immunization coverage with quality and safety, improve vaccine security and introduce newer vaccines based on epidemiological considerations [25], [26].

The UIP is implemented on the ground by nearly 200,000 Auxillary Nurse Midwives (ANMs) who vaccinate the children and pregnant women and are in turn supported by nearly a million Accredited Social Health Activists (ASHA) who counsel as well as mobilize the beneficiaries.

There is a strong monitoring and evaluation mechanism for the UIP at all levels. There is a regular reporting system from the health sub-center to primary health center (PHC), district, state and national level. This reporting has been computerized in the country as a part of a health management information system (HMIS), and the data are available from health facility level and above every month. Recently, the MoHFW has also implemented a reproductive child health portal to track every pregnant woman, mother and child up to 5 years of age to ensure delivery of health services. A review mechanism is established at all levels of programme implementation in the country. At PHC level it is through monthly meetings with ANMs and ASHAs, while at the district and state level it is via the District Task Force on Immunization (DTFI) as well as the State Task Force on Immunization (STFI). At National level the MoHFW has constituted an Immunization Action Group (IAG) to review the programme, discuss issues and suggest solutions at regular intervals.

#### 2.3.2 Training and capacity building for RVV introduction

For each phase of RVV introduction, training was imparted using a cascade model for each phase starting from a national level training of trainers (ToT) followed by state, district and sub-district level trainings. A national ToT workshop was conducted to establish a team of master trainers at the national level. The workshop was attended by national representatives of key immunization partners like WHO, UNICEF, JSI, GHS, and PATH. The workshop served as a platform not only to orient the master trainers but also to standardize the training material to be used by the master trainers for the subsequent state workshops. A standard set of slides and uniform messages were prepared to be used across all training workshops to ensure that the messages delivered were consistent at state, district, sub-district, and beneficiary levels. The standardized training package for RVV introduction included some key UIP approaches to open-vial/multi-dose vial policy, cold chain management, vaccine supply-chain management, session site management, as well as biomedical waste management with a specific focus on RVV. Similar state-level training workshops were conducted in all phase one and two states and over 400 healthcare officers were trained as state-level master trainers in each phase. The trainees for state level workshops included District Immunization Officers, Sub-district level Medical Officers and District Information, Education and Communication (IEC) Officers. All of the state workshops had a 100% attendance rate from the districts. The state workshops in the 9 states of phase 1 and phase 2 were followed by district and block level trainings to train more than 450,000 cold chain officers, front-line health workers, vaccinators and mobilisers. Thus, RVV introduction was used as a platform to reiterate key messages and approaches related to UIP with an aim to strengthen the overall routine immunization system.

The RVV being used in the nine phase one and phase two states is Rotavac® vaccine available for UIP in a 10 dose vial each vial consisting of 5 ml of vaccine. The vaccine is being given at 6, 10 and 14 weeks, 5 drops of vaccine (0.5 ml) administered orally at each visit. For operational feasibility, only the infants coming for the first dose of OPV and Pentavalent vaccine were started with RVV.

#### 2.3.3 Operations and cold chain management

Preparedness and gaps in the state health systems including AEFI surveillance system were assessed using a checklist developed by the MoHFW, Immunization Technical Support Unit and WHO. Immunization cards and related records/registers were updated to include RVV. Since RVV was not already included in the existing HMIS in first two phases, standardized formats for manual monthly reporting were developed. To apprise members about RVV and its potential association with intussusception, State and District AEFI Committee meetings were held before the RVV launch. Cold chain space availability was assessed using National Cold Chain Management Information System (NCCMIS) and Electronic Vaccine Intelligence System (eVIN) data.

#### 2.3.4 Communication and media advocacy

Standardized IEC materials were prepared for RVV introduction. Media workshops were conducted before the launch to ensure media engagement and facilitate positive messaging for RVV. With support from partner organizations, new vaccine introduction workshops were organized for civil society organizations in Odisha and Andhra Pradesh States seeking their support for a successful rollout. An RVV discussion platform was organized at the annual national conference of Indian Academy of Pediatrics (PEDICON 2016) to involve pediatricians in RVV discussion and rollout. A one-day orientation workshop on RVV introduction was organized for pediatricians working in the private and public sector in Odisha State.

### 2.4 Relevant changes

As stated, over 400 officers were trained at national and state level in both phases and more than 450,000 cold chain officers, front-line health workers, vaccinators and mobilisers were trained in nine introduction states. A deficit of about 15,000 L of storage space in 51 cold-chain points in phase I and 2 was mitigated through fast-track repair of non-functional equipment, re-appropriation of cold chain equipment and creation of satellite storage points. States prepared a detailed vaccine distribution plan with frequent supply cycles of small vaccine quantities to ensure that every session site would have at least one vial of RVV and that no point would receive more supply than it could store. Up until November 2017, a total of 13,260,000 RVV doses were administered in nine states (Table 1). As per HMIS data, form April 2016 until December 2017, a total of 329,625 AEFI cases were reported nationwide. Of these, causality assessment of 407 serious AEFI’s was done, of which 31 cases were assessed for RVV. However, in the causality assessments done no AEFI has be found to be directly associated with RVV which further establishes the safety of the vaccine [27].

**Table 1:** Cumulative coverage in children <12 months of age (in thousand doses administered and percentage coverage^a^) from date of introduction to November 2017 in nine Phase 1 and 2 states.

a The denominators for percentage coverage are derived from annual targets for each state from the date of start of RVV up to 30th November 2018.

## 3 Challenges and mitigation strategies during RVV introduction

### 3.1 Product related challenges

RVV (Rotavac®) is an oral vaccine with a confusingly similar presentation as Oral Polio Vaccine (OPV). Therefore, the workers were trained to differentiate RVV from OPV by the features like different color and size of droppers, as well as the positioning of vaccine vial monitors (VVM) on the cap for RVV (Rotavac®) versus on the vial for OPV. The manufacturer-instituted six-month expiry for Rotavac® at 2–8 °C proved to be unimplementable in the absence of any individual vial tracking technology. Based on data of six freeze–thaw cycles that RVV could withstand, the uniform cold chain storage and transport policy as guided by VVMs applicable for OPV was followed (with discard points for the VVM reached in 2 days at 37 °C and 225 days at <5 °C) [28].

### 3.2 Competing programme priorities

The introduction of RVV overlapped with several other major initiatives like Polio National Immunization Days and Sub-National Immunization Days, Mission Indradhanush (mission launched by GoI in 2014 to strengthen and re-energize the programme and achieve full immunization coverage for all children and pregnant women at a rapid pace), trivalent to bivalent OPV switch, and introduction of Inactivate Polio Vaccine [29]. However, the presence of national and state government leadership, clear distribution of roles to partners with allocation of resources and milestones, and establishment of dedicated RVV management cells at state levels by John Snow Inc (JSI) to provide catalytic support facilitated a successful seamless introduction with timely mitigation of challenges.

## 4 Conclusions and lessons learned

RVV introduction in India is a landmark achievement, and an illustration of GoI stewardship in terms of planning, funding, implementation and monitoring. This is the first time that a novel low-cost indigenous vaccine was introduced in the country with domestic funds. Moreover, introduction was supported by scientific evidence on efficacy, safety and programme preparedness. RVV introduction also provided an opportunity to re-evaluate gaps in the system and develop immediate and long-term plans to address them. The MoHFW assigned specific roles and responsibilities to partners allowing successful introduction within a short timeline alongside competing priorities. The third phase of the vaccine introduction has been started in June 2018 the vaccine for which was procured through Gavi support. However, the implementation and roll out of the vaccine will be funded by MoHFW, GoI, with technical assistance from the partners. The well-defined plan and meticulous implementation with support from various stakeholders ensured that the introduction was smooth and did not add additional burden on the programme. The fact that there were pre-assessments done to identify the gaps for each component of UIP allowed for timely mitigation of loop holes which benefited the programme in totality. This ensured that the RVV introduction strengthened the programme rather than burdening it. This is further supported by the fact that the MoHFW, GoI is committed to expand RVV to the entire country by the end of 2019. The experience of RVV introduction in India may be used to inform countries who might be looking for low-cost yet effective options.

## Authors’ contribution

Akash Malik, Arindam Ray, Bhrigu Kapuria, Sheenu Chaudhary, Pradeep Haldar, Robert Steinglass and Rakesh Kumar were involved in planning, implementation and monitoring of Rotavirus Vaccine introduction in India. Akash Malik, Arindam Ray, Bhrigu Kapuria, Sheenu Chaudhary were involved in compiling and analysis of the data generated from preparedness assessment and coverage data after vaccine was introduced. Akash Malik, Arindam Ray, Bhrigu Kapuria, Sheenu Chaudhary, Pradeep Haldar were involved in training of the master trainers at the national and state level. Akash Malik, Arindam Ray, Anita Seth and Mathuram Santosham conceptualized the manuscript, evaluated the available evidence and literature and prepared the drafts for review. All authors were involved in reviewing and finalizing the manuscript draft before submission.’

## Declaration of Competing Interest

The authors declare that they have no known competing financial interests or personal relationships that could have appeared to influence the work reported in this paper.
